# Flesh Quality Loss in Response to Dietary Isoleucine Deficiency and Excess in Fish: A Link to Impaired Nrf2-Dependent Antioxidant Defense in Muscle

**DOI:** 10.1371/journal.pone.0115129

**Published:** 2014-12-16

**Authors:** Lu Gan, Wei-Dan Jiang, Pei Wu, Yang Liu, Jun Jiang, Shu-Hong Li, Ling Tang, Sheng-Yao Kuang, Lin Feng, Xiao-Qiu Zhou

**Affiliations:** 1 Animal Nutrition Institute, Sichuan Agricultural University, Chengdu, Sichuan, China; 2 Fish Nutrition and Safety Production University Key Laboratory of Sichuan Province, Sichuan Agricultural University, Chengdu, Sichuan, China; 3 Key Laboratory for Animal Disease-Resistance Nutrition of China Ministry of Education, Sichuan Agricultural University, Chengdu, Sichuan, China; 4 Animal Nutrition Institute, Sichuan Academy of Animal Science, Chengdu, Sichuan, China; University of Nordland, Norway

## Abstract

The present study explored the impact of dietary isoleucine (Ile) on fish growth and flesh quality and revealed a possible role of muscle antioxidant defense in flesh quality in relation to dietary Ile. Grass carp (weighing 256.8±3.5 g) were fed diets containing six graded levels of Ile (3.8, 6.6, 9.3, 12.5, 15.2 and 18.5 g/kg) for eight weeks. The results indicated that compared with Ile deficiency (3.8 g/kg diets) and excess (18.5 g/kg diets) groups, 9.3–15.2 g Ile/kg diet supplementations promoted fish growth and muscle fat deposition, whereas 6.6–15.2 g Ile/kg diets supplementation enhanced muscle nutrients (protein and total EAAs) deposition. Furthermore, muscle shear force, pH value, and hydroxyproline concentration were improved by 9.3–12.5, 9.3 and 9.3 g Ile/kg diet supplementations, respectively. However, muscle cooking loss, lactate content, and activities of cathepsin B and L were decreased by 6.6–15.2, 9.3–12.5, 9.3–12.5 and 9.3–15.2 g Ile/kg diet supplementations, respectively. Additionally, 6.6–15.2 and 6.6–12.5 g Ile/kg diet supplementations attenuated malondialdehyde and protein carbonyl contents, respectively. The activities of copper/zinc superoxide dismutase (Cu/Zn-SOD) and glutathione peroxidase (GPx), and glutathione content were enhanced by 6.6–9.3, 6.6–12.5 and 6.6–15.2 g Ile/kg diet supplementations, respectively. Moreover, the relative mRNA expressions of antioxidant enzymes, including Cu/Zn-SOD (6.6–12.5 g/kg diets) and GPx (12.5 g/kg diets), as well as antioxidant-related signaling molecules, including NF-E2-related factor 2 (Nrf2) (6.6–12.5 g/kg diets), target of rapamycin (6.6–12.5 g/kg diets), ribosomal S6 protein kinase 1 (9.3–12.5 g/kg diets) and casein kinase 2 (6.6–12.5 g/kg diets), were up-regulated when Ile diet supplementations were administered at these levels, respectively, whereas the relative mRNA expression of Kelch-like ECH-associated protein 1 was down-regulated with 9.3 g Ile/kg diet supplementations. Collectively, the present study indicated that optimum isoleucine improved flesh quality, partly due to the activation of antioxidant defense through the Nrf2 signaling pathway.

## Introduction

Between 1980 and 2010, the global per capita consumption of fish increased by more than 58% with the rapid development of aquaculture [1]. However, flesh quality deterioration is one of the most important issues in the aquaculture industry [2], which leads to poor consumption [3], increases the risk of cardiovascular disease in consumers [4] and causes tremendous economic losses for producers [5]. Hence, it is necessary to address this issue of fish flesh quality.

Isoleucine (Ile), one type of branched-chain amino acids, is an essential nutrient of all fish species studied and primarily deposited in body protein, notably in skeletal muscle protein [6]. Remarkably, Ile accounts for nearly 4.2% of total muscle protein in grass carp (*Ctenopharyngodon idellus*) [7]. To the best of our knowledge, there is no information concerning the effect of Ile on flesh quality of fish. Different from non-branched-chain amino acids, Ile largely escapes first-pass hepatic catabolism and is catabolized primarily in skeletal muscle leading to the formation of glutamate [6]. A recent study on Atlantic salmon (*Salmo salar* L.) showed that dietary glutamate supplementation enhanced fillet firmness [8]. Moreover, dietary Ile supplementation reduced salivary cortisol level in human during intense endurance exercise [9]. Acerete et al. [10] reported that high plasma cortisol levels might be responsible for flesh deterioration in European sea bass (*Dicentrarchus labrax*). These findings indicated a likely correlation between Ile and fish flesh quality, which warrants investigation.

The content of polyunsaturated unsaturated fatty acids (PUFA) of total fatty acids in grass carp muscle is approximately 33% [7], which is higher than that of pigs (approximately 20%) [11]. Nevertheless, an abundance of PUFA in fish muscle is susceptible to oxidative damage caused by exceeding reactive oxygen species (ROS) [12]. ROS-induced oxidative damage is considered to be a major factor leading to quality deterioration of fish muscle [13]. Generally, ROS including superoxide anion (O_2_^•−^), hydrogen peroxide (H_2_O_2_) and hydroxyl radical (•OH), are produced in fish, primarily as a result of aerobic metabolism in mitochondria [14]. However, no information is reported regarding the relationship between Ile and ROS in fish muscle. In *Micrococcus luteus* cells, Ile could chelate metal ions through its non-protonated amino group [15]. α-keto-β-methyl-*n*-valeric acid (KMV), a metabolite of isoleucine, was shown to be a scavenger of ROS in an *in* *vitro* assay [16]. These data indicated that Ile might influence ROS accumulation in fish. However, whether Ile can affect flesh quality by enhancing the scavenging ability of ROS in fish muscle is unknown, awaiting additional investigation.

Generally, the ROS removal in fish is achieved through antioxidant defense, which in large part relies on the antioxidant substances, such as glutathione (GSH) and antioxidant enzyme (superoxide dismutase, SOD; catalase, CAT; glutathione peroxidase, GPx) activities [12]. The rate-limiting step of GSH biosynthesis is performed by glutamate cysteine ligase (GCL) [17]. Additionally, the activities of antioxidant enzymes may be associated with their gene transcription levels. In rat skeletal muscle, studies have revealed that the elevation of Cu/Zn-SOD, CAT and GPx activities likely occurred, in part, due to an increase in mRNA levels [18]. To date, little information is available regarding the effect of Ile on antioxidant enzyme gene expression. The expression of antioxidant enzyme gene is typically regulated by Nrf2 (NF-E2-related factor 2)-Keap1 (kelch-like ECH-associated protein 1) signaling in fish [19]. Furthermore, Nrf2 can be activated by its upstream signaling molecules, such as TOR (the target of rapamycin) in mice liver [20]. Moreover, CK2 (casein kinase 2) emerged as an ubiquitous cellular signaling molecule and stimulated TOR expression in human lung carcinoma cells [21]. Recently, we have been firstly cloned the cDNA of Nrf2 (Genbank Accession No. KF733814), Keap1 (Genbank Accession No. KF811013), TOR (Genbank Accession No. JX854449) and CK2 (Genbank Accession No. KF914143) in grass carp. To date, information has been scarce concerning the effects of Ile on these signaling molecules of the Nrf2 pathway in fish. Ile can promote insulin secretion in dogs [22]. Several reports have demonstrated that insulin activated TOR signaling pathway in rainbow trout (*Oncorhynchus mykiss*) [23] and simulated CK2 expression in 3T3-L1 adipocytes [24]. These findings indicated that a likely correlation exists between Ile and signaling molecules involved in the Nrf2 pathway in fish muscle, which requires additional exploration.

Grass carp, with good taste and high nutritional value, is cultured worldwide [25]. The world aquaculture production of grass carp was 4,574,673 tons and ranked second among cultured fish production in 2011 [1]. Presently, with the rapid development of commercial rearing of fish, the use of formulated feed is necessary. The formulation of such feeds is, in large part, dependent on complete information of the nutritional requirements of this species [25]. The Ile requirement for growth was estimated in juvenile grass carp [26]. However, several studies have implied that different response criteria (flesh firmness and fish growth) [8] and growth stages [27] may result in different amino acid requirements. Hence, it is essential to determine the Ile requirements for flesh quality and growth in young grass carp.

The present study investigated the effects of Ile on flesh quality in fish and revealed a likely role of antioxidant defense on flesh quality in relation to dietary Ile. Furthermore, expressions of antioxidant enzymes and signaling molecules involved in the Nrf2 pathway were measured to explore the potential way of Ile-mediated antioxidant defense. The present data aimed to evaluate the effect of Ile on fish flesh quality, as well as the optimum dietary Ile levels, for flesh quality and growth in young grass carp.

## Materials and Methods

### Ethics statement

All experimental protocols were approved by the Animal Care Advisory Committee of Sichuan Agricultural University.

### Experimental design and diets

The formulation of the basal diet is shown in Table 1. Fish meal, casein and gelatin were used as the primary protein sources. The dietary protein level fixed at 300 g/kg was reported to be optimal for promoting the growth of grass carp [25]. L-isoleucine was added to the basal diet to provide graded concentrations of 4.0 g Ile/kg (control), 7.0 g Ile/kg, 10.0 g Ile/kg, 13.0 g Ile/kg, 16.0 g Ile/kg, and 19.0 g Ile/kg diet. The diets were made isonitrogenous by adjusting crystalline L-glycine according to the method of Chen et al. [28]. The pellets were produced and stored at −20°C until they were used according to the methods described by Lin et al. [29]. Using high-performance liquid chromatography (HPLC), as described by Wu and Mao [7], the Ile concentrations in diets were analyzed to be 3.8 g/kg, 6.6 g/kg, 9.3 g/kg, 12.5 g/kg, 15.2 g/kg and 18.5 g/kg diet.

**Table 1:** The composition and nutrient content of the basal diet.

Ingredients	g/kg	Nutrient content^1^	g/kg
Fish meal	78.0	Crude protein	309.6
Casein	30.0	Crude lipid	49.2
Gelatin	39.9	Available phosphorus	6.0
Crystalline AA mix^2^	154.8	n-3	10.0
Isoleucine premix^3^	50.0	n-6	10.0
Glycine premix^4^	80.0		
α-starch	280.0		
Corn starch	87.2		
Fish oil	22.0		
Soybean oil	18.9		
Vitamin premix^5^	10.0		
Trace mineral premix^6^	20.0		
Ca (H_2_PO4)_2_	22.7		
Choline chloride (500 g/kg)	6.0		
Microcrystalline cellulose	100.0		
Ethoxyquin (300 g/kg)	0.5		

1 Crude protein and crude lipid contents were measured value. Available phosphorus, n-3 and n-6 contents were calculated value.

2 Amino acid mix (g/kg): lysine, 17.13 g; methionine, 7.78 g; tryptophan, 3.57 g; threonine 11.88 g; arginine, 12.89 g; histidine, 7.96 g; leucine, 20.51 g; phenylalanine, 13.60 g; valine, 15.33 g; cystine, 0.91 g; tyrosine, 10.86 g; glutamic acid, 32.32 g.

3 L-isoleucine was added to obtain graded levels of isoleucine. Per kilogram of isoleucine premix composition from diet 1 to 6 was as follows (g/kg): L-isoleucine 0.00, 60.86, 121.71, 182.57, 243.42, 304.28 g and corn starch 1000.00, 939.14, 878.29, 817.43, 756.58, 695.72, respectively.

4 Glycine premix: Each mixture was made isonitrogenous with the addition of reduced amounts of glycine and compensated with appropriate amounts of corn starch. Per kilogram of glycine premix composition from diet 1 to 6 was as follows (g/kg): 720.96, 699.49, 678.03, 656.57, 635.10, 613.64 g and corn starch 279.04, 300.51, 321.97, 343.43, 364.90, 386.36 g, respectively.

5 Per kg of vitamin premix (g/kg): retinyl acetate (500 000 IU/g), 0.80 g; cholecalciferol (500 000 IU/g), 0.48 g; DL-α-tocopherol acetate (500 g/kg), 20.00 g; menadione (230 g/kg), 0.22 g; cyanocobalamin (10 g/kg), 0.10 g; D-biotin (20 g/kg), 5.00 g; folic acid (960 g/kg), 0.52 g; thiamine hydrochloride (980 g/kg), 0.12 g; ascorhyl acetate (930 g/kg), 7.16 g; niacin (990 g/kg), 2.58 g; meso-inositol (990 g/kg), 52.33 g; calcium-D-pantothenate (900 g/kg), 2.78 g; riboflavin (800 g/kg), 0.99 g; pyridoxine hydrochloride (980 g/kg), 0.62 g. All ingredients were diluted with corn starch to 1 kg.

6 Per kg of trace mineral premix (g/kg): FeSO_4_.H_2_O, 25.00 g; CuSO_4_.5H_2_O, 0.60 g; ZnSO_4_.H_2_O, 4.35 g; MnSO_4_.H_2_O, 2.04 g; KI, 1.10 g; NaSeO_3_, 2.50 g; MgSO4.H_2_O, 230.67 g. All ingredients were diluted with corn starch to 1 kg.

### Fish Trial

The young grass carp were obtained from an aquaculture stock (Sichuan, China). All of the fish were adapted to the experimental conditions for 2 weeks according to Ahmed and Khan [30]. A total of 540 fish with an average weight of 256.8 (SD, 3.5) g were randomly distributed into 18 (measuring 1.4 m×1.4 m×1.4 m) cages; each cage was equipped with a disc of 100-cm diameter in the bottom to collect the uneaten food, as described by Wu et al. [31]. The treatments were randomly assigned to 3 cages. The fish were fed to apparent satiation 4 times per day for 8 weeks according to Tang et al. [32]. The dissolved oxygen was higher than 6 mg/L. Water temperature and pH were maintained at 26±2°C and 7.0±0.5, respectively. The photoperiod was adjusted to ambient light regime as previously described by Olsen et al. [33].

### Sample collection and analysis

At the initiation and termination of the feeding trial, the fish in each cage were weighed. After an 8-week feeding Trial, six fish from each treatment group were collected; blood samples were obtained from the caudal vein with heparinized syringes; the blood samples were used for plasma ammonia determination according to the method described by Chen et al [28]. Other fish from each treatment group were anaesthetized (MS 222 metacaine) as described by Larsson et al. [8] and were subsequently killed by a blow to the head according to Liu et al. [34]. The muscle samples were selected as described by Hosseini et al. [4]. The fish were manually filleted, using a new sterile scalpel for each fish. The left sides of the filleted fish were immediately frozen in liquid nitrogen and were stored at −70°C for proximate composition and antioxidant status; and the other side of the fish was used for determination of flesh quality parameters. Moisture (oven drying to a constant weight), crude protein (N-Kjeldahl×6·25), crude fat (solvent extraction with petroleum) were measured according to the methods of Khan et al. [25]. The method for determining muscle amino acid composition was similar to that for determining Ile diet. The shear force required to puncture the fillet surface was recorded to express muscle firmness as previously described by Bahuaud et al. [35]. Cooking loss and hydroxyproline content were analyzed as reported in a previous study by Kong et al. [36]. Cooking loss was calculated as the percent weight reduction of the cooked sample compared with the raw sample to determine muscle water holding capacity (WHC), whereas hydroxyproline content was measured by hydrolysis in hydrochloric acid to determine muscle collagen content. Muscle pH value was detected according to Acerete et al. [10], with slight modifications. The pH value was directly read in the muscle using a needle pH meter. Additionally, cathepsin B and L activities were measured using the fluorimetrical method according to Bahuaud et al. [35], with slight modifications. Benzyloxycarbonyl-arginylarginine-4-methyl-7-coumarylamide (Z-Arg-Arg-MCA) for cathepsin B activity and benzyloxycarbonyl-L-phenylalany-L-arginyl-4-methyl-7-coumarylamide (Z-Phe-Arg-MCA) for cathepsin L activity were used as substrates. Lactate content was measured using enzymatic colorimetric analysis according to Acerete et al. [11]. The malondialdehyde (MDA) content was investigated using the thiobarbituric acid reaction according to the method of Li et al. [37]. The protein carbonyl (PC) content was measured using 2,4-dinitrophenylhydrazine (DNPH) reagent, as described by Jiang et al. [38]. The anti-superoxide anion (ASA) capacity was measured using the griess reagent and the anti-hydroxyl radical (a-HR) capacity analysed using the Fenton reaction, which was referenced by the method of Wu et al. [31]. The activities of SOD, GPx and CAT were detected according to Lambertucci et al. [18]. The SOD activity was determined by measuring the decrease in the rate of cytochrome c reduction in a xanthine-xanthine oxidase superoxide generating system. The GPx activity was determined by measuring the rate of NADPH oxidation. The CAT activity was determined by measuring the decomposition of hydrogen peroxide. The GSH content was determined by measuring the formation of 5-thio-2-nitrobenzoate (TNB) according to the method of Tang et al. [32].

### Real-time PCR analysis

The procedures of RNA isolation, reverse transcription and quantitative real-time PCR were similar to those previously described by Bahuaud et al. [35]. The total RNA was extracted from muscle using an RNAiso plus kit. The RNA quantity and quality were assessed using spectrophotometry. Subsequently, the first-strand cDNA was synthesized using a PrimeScripte RT reagent Kit, according to the manufacturer’s instructions. The quantitative real-time PCR was performed on a CFX96 Real-Time PCR Detection System using Prime Script RT-PCR Kit II. Specific primers for GCL, Nrf2, Keap1, TOR and CK2 genes were designed according to the grass carp sequences cloned in our laboratory, and primers for Cu/Zn-SOD, CAT, GPx, and S6K1 genes were designed using the published sequences of grass carp. The primer sequences and optimal annealing temperatures are shown in Table 2. All of the primer amplification efficiencies were approximately 100% for these genes. β-actin expression was the choice for normalization based on the results of our preliminary experiment regarding the evaluation of internal control genes (S2 and S3 Tables).

**Table 2:** Forward (F) and reverse (R) primers used for quantitative PCR, including amplicon length and annealing temperature.

Gene	Description	Primer	Annealing temperature (°C)	Genbank accession no.
*Cu/Zn-SOD*	copper/zinc superoxide dismutase	F: CGCACTTCAACCCTTACA R: ACTTTCCTCATTGCCTCC	61.5	GU901214
*CAT*	Catalase	F: AAGTTCTACACCGATGAGG R: CCAGAAATCCCAAACCAT	58.7	FJ560431
*GPx*	Glutathione peroxidase	F: GGGCTGGTTATTCTGGGC R: AGGCGATGTCATTCCTGTTC	61.5	EU828796
*GCL*	Glutamate-cysteine ligase	F: CACGCTGCCAGAATACAA R: ATCACCACCTTTTCGCC	56.9	KF998103
*Nrf2*	NF-E2-related factor 2	F: CTGGACGAGGAGACTGGA R: ATCTGTGGTAGGTGGAAC	62.5	KF733814
*Keap1*	Helch-like ECH-associated protein 1	F: TTCCACGCCCTCCTCAA R: TGTACCCTCCCGCTATG	63.0	KF811013
*TOR*	Target of rapamycin	F: TCCCACTTTCCACCAACT R: ACACCTCCACCTTCTCCA	61.4	JX854449
*S6K1*	Ribosomal S 6 protein kinase1	F: TGGAGGAGGTAATGGACG R: ACATAAAGCAGCCTGACG	54.0	EF373673
*CK2*	Casein kinase 2	F: CCCCAACCACAGTGACCT R: TCCCTGCTGATACTTCTCC	57.9	KF914143
*β-Actin*		F: GGCTGTGCTGTCCCTGTA R: GGGCATAACCCTCGTAGAT	61.4	M25013

### Statistical analyses

The data were analyzed using one-way analysis of variance (ANOVA), and the Duncan method was subsequently employed to determine significant differences among the treatment groups at the level of *P*<0.05 using SPSS 18.0 (SPSS Inc., Chicago, IL, USA). The correlations were obtained using the Pearson correlation coefficients, at the same level of significance. The relationship between dietary Ile and growth performance, muscle composition, flesh quality parameters as well as muscle antioxidant parameters were subjected to a quadratic regression model as described by Tang et al. [32]. A quadratic regression model was used to estimate dietary Ile requirements based on muscle cooking loss and PWG according to Chen et al. [28].

## Results

### Growth performance and muscle composition

**Figure 1:**
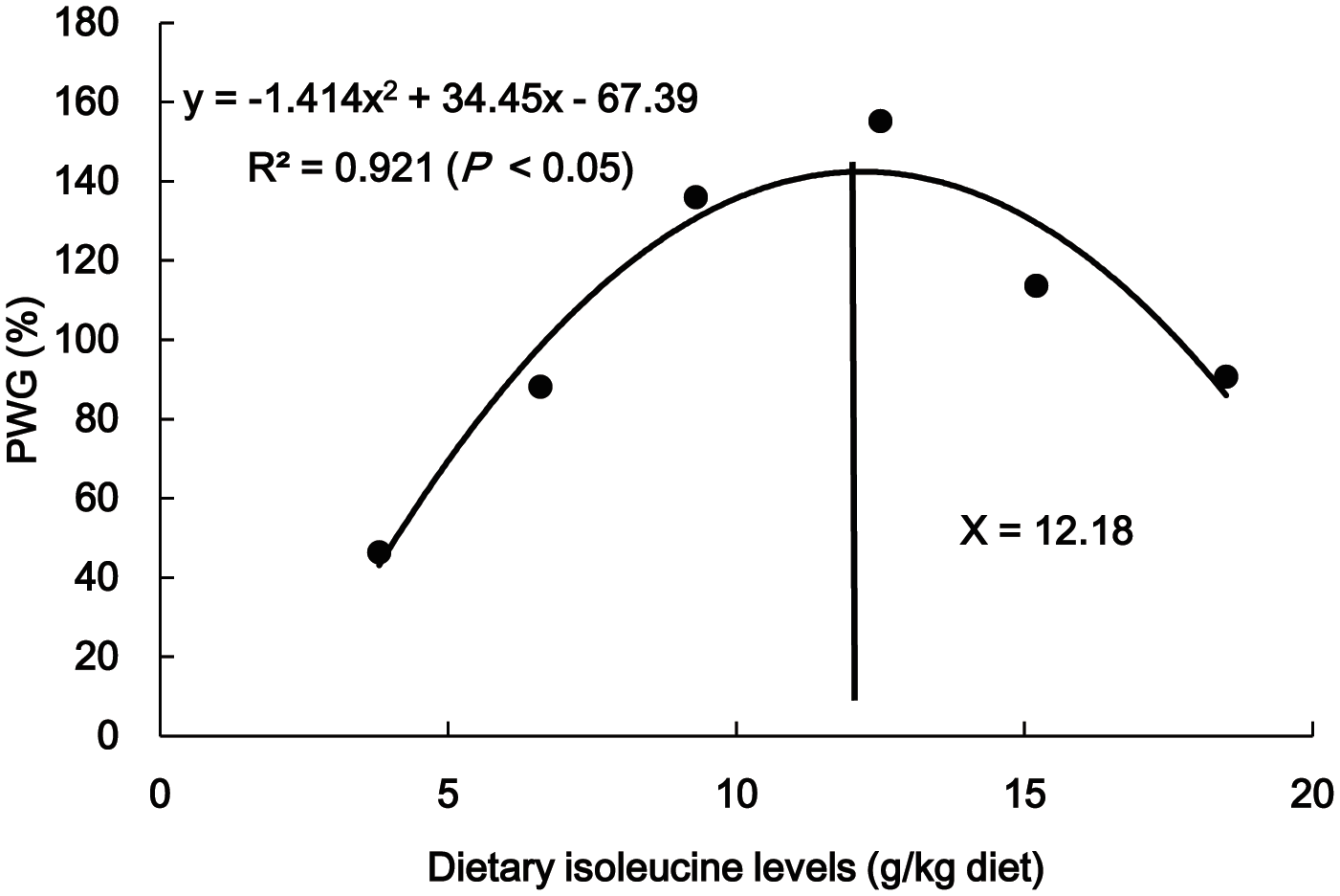
Quadratic regression analysis of PWG for grass carp fed graded levels of Ile. PWG, percentage weight gain.

Growth performance factors, PAC and muscle proximate composition are presented in Table 3. FBW, PWG, SGR, FE and PER gradually increased with higher Ile levels up to 12.5 g/kg diet and then they significantly decreased (*P*<0.05). FI improved as dietary Ile levels increased from 3.8 g/kg to 9.3 g/kg diet (*P*<0.05) and decreased thereafter (*P*<0.05). PAC decreased with higher Ile levels up to 9.3 g/kg diet, and increased thereafter (*P*<0.05). Fish that were fed 12.5 g Ile/kg diet had lower muscle moisture content than fish that were fed 3.8 g Ile/kg diet (*P*<0.05), and no significant differences were found among the other groups (*P*>0.05). The protein and lipid contents increased with higher levels of dietary Ile up to 12.5 g/kg diet and decreased thereafter (*P*<0.05). Additionally, FBW, PWG, SGR, FI, FE, PER, and PAC as well as muscle moisture, protein and fat contents showed a quadratic response to dietary Ile levels (Table 3). As shown in Fig. 1, the dietary Ile requirement of young grass carp (253.4–660.8 g) estimated by the quadratic regression analysis based on PWG was 12.2 g/kg diet, corresponding to 39.3 g/kg of dietary protein.

**Table 3:** Treatment effects on growth performance factors, plasma ammonia content (µmol/L) and muscle proximate composition (%) of grass carp.

Dietary isoleucine levels (g/kg diet)						
	3.8	6.6	9.3	12.5	15.2	18.5
IBW^1^	255.7±4.5^a^	253.4±5.1^a^	257.1±2.3^a^	258.9±2.4^a^	257.3±1.8^a^	258.4±3.7^a^
FBW^1^	374.4±4.0^a^	476.5±6.7^b^	606.4±11.1^d^	660.8±18.3^e^	549.6±8.7^c^	492.5±8.0^b^
PWG^1^	46.42±1.15^a^	88.11±4.62^b^	135.93±6.47^d^	155.24±8.50^e^	113.59±4.01^c^	90.65±5.23^b^
SGR^1^	0.681±0.014^a^	1.128±0.044^b^	1.532±0.049^d^	1.673±0.060^e^	1.355±0.034^c^	1.152±0.049^b^
FI^1^	282.6±1.1^a^	368.5±3.5^b^	586.5±0.7^f^	563.4±1.1^e^	488.2±0.9^d^	418.3±1.0^c^
FE^1^	41.99±0.37^a^	60.57±2.79^b^	59.56±2.24^b^	71.33±3.35^c^	59.86±1.79 ^b^	55.97±2.55^b^
PER^1^	1.356±0.012^a^	1.956±0.090^c^	1.924±0.072^bc^	2.304±0.108^d^	1.934±0.058^bc^	1.808±0.082^b^
PAC^2^	532.6±21.5^f^	297.4±16.1^c^	221.3±20.2^a^	249.4±18.2^b^	332.4±23.5^d^	359.9±22.8^e^
Moisture^2^	76.99±1.62^b^	76.68±0.78^ab^	76.02±0.72^ab^	75.65±0.68^a^	75.93±0.64^ab^	76.73±0.64^ab^
Protein^2^	17.21±0.43^a^	18.18±0.66^b^	18.53±0.55^bc^	18.97±0.45^c^	18.82±0.57^bc^	17.24±0.72^a^
Lipid^2^	2.472±0.303^ab^	2.686±0.162^bc^	2.788±0.107^c^	3.079±0.123^d^	2.875±0.252^cd^	2.289±0.067^a^
Regression						
Y_PWG_ = −1.414×^2^+34.448x−67.383				R^2^ = 0.921		*P*<0.05
Y_SGR_ = −0.013×^2^+0.314x−0.334				R^2^ = 0.949		*P*<0.05
Y_FI_ = −4.054×^2^+99.714x−53.368				R^2^ = 0.863		*P* = 0.051
Y_FE_ = −0.320×^2^+7.871x+18.316				R^2^ = 0.822		*P* = 0.075
Y_PER_ = −0.010×^2^+0.254x+0.591				R^2^ = 0.823		*P* = 0.075
Y_PAC_ = 3.970×^2^−95.134x+798.684				R^2^ = 0.827		*P* = 0.072
Y_moisture_ = 0.020×^2^−0.476x+78.684				R^2^ = 0.893		*P*<0.05
Y_protein_ = −0.031×^2^+0.715x+14.844				R^2^ = 0.930		*P*<0.05
Y_lipid_ = −0.011×^2^+0.244x+1.624				R^2^ = 0.841		*P* = 0.063

1 Mean values of triplicate groups, with thirty fish in each group. And mean values within the same row with different superscripts are significantly different (*P*<0.05).

2 Values are means ± SD (n = 6), and mean values within the same row with different superscripts are significantly different (*P*<0.05).

IBW: initial body weight (g/fish); FBW: final body weight (g/fish); PWG: percentage weight gain (%); SGR: specific growth rate (%/day); FI: feed intake (g/fish); FE: feed efficiency (%); PER: protein efficiency ratio; PAC: plasma ammonia content.

weight gain (WG) = FBW (g)–IBW (g);

PWG = 100×WG (g)/IBW (g);

SGR = 100×[ln FBW (g)-ln IBW (g)]/number of days;

FE = 100×weight gain (g)/feed intake (g);

PER = wet weight gain (g)/protein intake (g).

### Amino acid composition of muscle

The amino acid composition of grass carp muscle fed diets with graded levels of Ile is shown in Table 4. The Ile concentration in muscle exhibited a second-order polynomial curve (Y = 0.0002x^2^+0.0154x+3.17, R^2^ = 0.938, *P*<0.05) respect to dietary Ile levels, suggesting that the Ile concentration in fish muscle gradually increased with dietary Ile supplementation. Aspartic acid, threonine, serine, glutamic acid, glycine, alanine, methionine, leucine, tyrosine and lysine contents increased with higher dietary Ile levels and subsequently decreased with increasing dietary Ile levels up to 18.5 g/kg diet (*P*<0.05). However, valine, cysteine, phenylalanine, histidine and arginine contents were not significantly affected by dietary Ile (*P*>0.05).

**Table 4:** Treatment effects on amino acid composition (% Dry Weight) in grass carp muscle.

Dietary isoleucine levels (g/kg diet)						
	3.8	6.6	9.3	12.5	15.2	18.5
Ile	3.25±0.01^a^	3.27±0.02^a^	3.29±0.01^a^	3.43±0.02^b^	3.46±0.02^bc^	3.50±0.03^c^
Asp	7.66±0.01^a^	7.96±0.04^b^	7.87±0.03^b^	7.95±0.03^b^	7.97±0.05^b^	7.62±0.06^a^
Thr	3.46±0.03^a^	3.63±0.03^b^	3.58±0.07^b^	3.63±0.03^b^	3.57±0.01^b^	3.43±0.03^a^
Ser	3.26±0.01^a^	3.42±0.03^b^	3.44±0.06^b^	3.45±0.01^b^	3.40±0.06^b^	3.20±0.02^a^
Glu	13.84±0.12^a^	14.54±0.14^b^	14.37±0.03^b^	14.50±0.03^b^	14.49±0.12^b^	13.80±0.06^a^
Gly	3.80±0.06^ab^	4.01±0.02^bc^	4.18±0.01^c^	4.05±0.01^c^	4.06±0.22^c^	3.76±0.04^a^
Ala	4.44±0.14^a^	4.72±0.01^c^	4.56±0.07^b^	4.67±0.01^ab^	4.66±0.04^ab^	4.37±0.01^a^
Val	3.68±21.5^a^	3.71±0.16^a^	3.59±0.02^a^	3.72±0.05^a^	3.71±0.08^a^	3.57±0.10^a^
Cys	0.50±0.04^a^	0.49±0.05^a^	0.53±0.01^a^	0.45±0.09^a^	0.51±0.03^a^	0.40±0.07^a^
Met	2.20±0.03^ab^	2.30±0.03^c^	2.28±0.03^bc^	2.28±0.06^bc^	2.24±0.02^bc^	2.13±0.04^a^
Leu	6.17±0.04^a^	6.47±0.08^b^	6.38±0.01^b^	6.49±0.03^b^	6.41±0.01^b^	6.19±0.05^a^
Tyr	2.80±0.05^a^	3.00±0.09^c^	2.93±0.04^bc^	2.99±0.03^c^	2.95±0.02^c^	2.82±0.03^ab^
Phe	3.16±0.08^a^	3.32±0.13^a^	3.20±0.02^a^	3.33±0.03^a^	3.28±0.08^a^	3.14±0.05^a^
Lys	6.80±0.22^ab^	7.03±0.23^bc^	7.10±0.12^bc^	7.04±0.04^bc^	7.30±0.17^c^	6.56±0.06^a^
His	1.63±0.03^a^	1.84±0.05^a^	2.02±0.32^a^	1.82±0.04^a^	1.86±0.22^a^	1.60±0.07^a^
Arg	5.16±0.33^a^	5.21±0.07^a^	5.14±0.05^a^	5.20±0.16^a^	5.15±0.02^a^	5.41±0.34^a^
Total EAA	35.51±0.28^a^	36.78±0.11^b^	36.57±0.39^b^	36.94±0.18^b^	36.98±0.56^b^	35.55±0.35^a^

Values are means ± SD (n = 6), and mean values within the same row with different superscripts are significantly different (*P*<0.05).

### Flesh quality parameters

**Figure 2:**
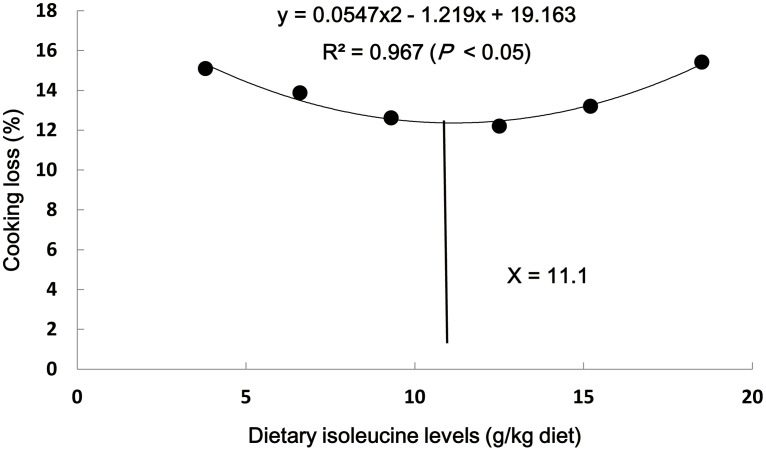
Quadratic regression analysis of muscle cooking loss for grass carp fed graded levels of Ile.

As shown in Table 5, dietary Ile has significant effects on flesh quality parameters of grass carp. Cooking loss was the highest for fish that were fed 3.8 g Ile/kg and 18.5 g Ile/kg diets (*P*<0.05). The shear force increased with higher dietary Ile levels up to 9.3 g/kg diet and subsequently decreased (*P*<0.05). The hydroxyproline content followed a similar pattern to that observed with shear force. The pH value elevated with increasing dietary Ile levels up to 9.3 g/kg diet and gradually decreased thereafter (*P*<0.05). The lactate content exhibited an opposite pattern of pH. The cathepsin B and L activities decreased with increasing dietary Ile levels up to 9.3 g/kg diet and subsequently gradually increased (*P*<0.05). The regression analysis showed a quadratic relationship between muscle cooking loss, shear force, pH, cathepsin B and L activities and lactate content with dietary Ile levels (Table 5). On subjecting the muscle cooking loss and dietary Ile levels to quadratic regression analysis, optimum leucine level was found at 11.1 g/kg diet or 36.0 g/kg of dietary protein (Fig. 2).

**Table 5:** Treatment effects on muscle cooking loss (%), shear force (N), hydroxyproline content (µg/mg wet tissue), pH value, cathepsin activities (U/g muscle) and lactate content (mmol/g protein) in grass carp.

Dietary isoleucine levels (g/kg diet)						
	3.8	6.6	9.3	12.5	15.2	18.5
Cooking loss	15.09±1.22^c^	13.87±1.25^b^	12.62±0.81^a^	12.20±0.98^a^	13.20±0.30^ab^	15.42±0.66^c^
Shear force	1.855±0.119^ab^	1.958±0.098^bc^	2.232±0.156^d^	2.022±0.152^c^	1.972±0.123^bc^	1.723±0.118^a^
Hydroxyproline	0.240±0.021^bc^	0.247±0.022^c^	0.327±0.012^d^	0.263±0.022^c^	0.222±0.017^ab^	0.203±0.016^a^
pH	5.992±0.028^a^	6.045±0.034^bc^	6.080±0.029^c^	6.050±0.053^bc^	6.035±0.040^abc^	6.033±0.019^ab^
Cathepsin B	5.923±0.642^b^	5.445±0.479^b^	4.420±0.385^a^	4.697±0.518^a^	5.644±0.545^b^	5.838±0.502^b^
Cathepsin L	1.218±0.130^b^	1.138±0.121^b^	0.991±0.100^a^	1.000±0.091^a^	1.093±0.100^ab^	1.114±0.083^b^
Lactate	4.146±0.355^d^	3.829±0.228^c^	2.269±0.169^a^	2.476±0.216^a^	3.504±0.245^b^	3.950±0.274^cd^
Regression						
Y_cooking loss_ = 0.115×^2^−3.108x+33.402				R^2^ = 0.854		*P* = 0.056
Y_shear force_ = −0.006×^2^+0.13x+1.440				R^2^ = 0.830		*P* = 0.070
Y_pH_ = −0.001×^2^+0.023x+5.930				R^2^ = 0.684		*P* = 0.177
Y_cathepsin B_ = 0.023×^2^−0.499x+7.501				R^2^ = 0.738		*P* = 0.134
Y_cathepsin L_ = 0.003×^2^−0.073x+1.456				R^2^ = 0.824		*P* = 0.074
Y_Lactate_ = 0.029×^2^−0.66x+6.385				R^2^ = 0.745		*P* = 0.128

Values are means ± SD (n = 6), and mean values within the same row with different superscripts are significantly different (*P*<0.05).

### Muscle antioxidant parameters

The effects of dietary Ile supplementation on muscle antioxidant parameters of grass carp are presented in Table 6. The lowest PC content was obtained for fish that were fed diets containing 9.3 g Ile/kg diet (*P*<0.05), whereas the fish that were fed diets containing 15.2 g Ile/kg diet had the lowest MDA content. ASA and a-HR capacities increased as dietary Ile levels rose up to 6.6 g Ile/kg and 9.3 g Ile/kg diet, respectively, and decreased with increasing dietary Ile levels up to 15.2 g Ile/kg diet (*P*<0.05), at which point the response reached a plateau (*P*>0.05). The Cu/Zn-SOD activity increased with increasing dietary Ile levels up to 9.3 g/kg diet and decreased with Ile levels further increasing (*P*<0.05). The GPx activity was the highest for fish that were fed a diet containing 9.3 g Ile/kg diet (*P*<0.05). The CAT activity was the highest for fish that were fed with 3.8 g Ile/kg diet and lowest for fish that were fed a diet containing 12.5 g Ile/kg diet (*P*<0.05). The GSH content was significantly improved with increasing dietary Ile levels up to 12.5 g Ile/kg diet and decreased thereafter (*P*<0.05). The regression analysis showed that these parameters quadratically responded to dietary Ile levels (Table 6).

**Table 6:** Treatment effects on protein carbonyl (PC, nmol/mg protein), malondialdehyde (MDA, nmol/mg protein), anti-superoxide anion (ASA, U/g protein), anti-hydroxyl radical (a-HR, U/mg protein), copper/zinc superoxide dismutase (Cu/Zn-SOD, U/mg protein), glutathione peroxidase (GPx, U/mg protein), catalase (CAT, U/mg protein) activities and glutathione (GSH, mg/g protein) content in grass carp muscle.

Dietary isoleucine levels (g/kg diet)						
	3.8	6.6	9.3	12.5	15.2	18.5
PC	8.27±0.83^d^	4.40±0.52^b^	2.26±0.25^a^	4.47±0.50^b^	4.95±0.66^bc^	5.18±0.38^c^
MDA	1.54±0.12^d^	0.92±0.09^c^	0.60±0.06^b^	0.61±0.04^b^	0.36±0.04^a^	1.63±0.16^d^
ASA	29.71±1.99^c^	33.91±3.79^d^	30.42±1.27^c^	25.75±1.59^b^	18.95±0.63^a^	19.65±1.08^a^
a-HR	419.8±28.9^c^	415.0±21.5^c^	446.4±11.1^d^	381.7±11.6^b^	292.9±8.0^a^	297.5±29.3^a^
Cu/Zn-SOD	4.24±0.30^b^	5.43±0.53^c^	5.65±0.41^c^	4.12±0.37^b^	3.58±0.31^a^	3.31±0.20^a^
GPx	99.5±4.8^a^	113.5±8.6^b^	135.1±9.9^c^	119.4±10.2^b^	102.7±9.6^a^	97.9±7.3^a^
CAT	1.76±0.16^d^	1.37±0.13^c^	1.13±0.10^b^	0.97±0.07^a^	1.11±0.10^b^	1.19±0.08^b^
GSH	3.02±0.21^a^	5.99±0.56^c^	7.36±0.61^d^	7.94±0.65^e^	6.06±0.51^c^	4.49±0.17^b^
Regression						
Y_PC_ = 0.063×^2^−1.498x+12.264				R^2^ = 0.667		*P* = 0.192
Y_MDA_ = 0.021×^2^−0.469x+3.087				R^2^ = 0.859		*P* = 0.053
Y_ASA_ = −0.053×^2^+0.194x+31.923				R^2^ = 0.815		*P* = 0.080
Y_a-HR_ = −0.784×^2^+7.220x+410.426				R^2^ = 0.810		*P* = 0.083
Y_Cu/Zn-SOD_ = −0.020×^2^+0.325x+3.710				R^2^ = 0.683		*P* = 0.178
Y_GPx_ = −0.493×^2^+10.376x+69.181				R^2^ = 0.709		*P* = 0.157
Y_CAT_ = 0.008×^2^−0.217x+2.457				R^2^ = 0.976		*P*<0.01
Y_GSH_ = −0.074×^2^+1.711x−2.261				R^2^ = 0.958		*P*<0.01

Values are means ± SD (n = 6), and mean values within the same row with different superscripts are significantly different (*P*<0.05).

### Factors involved in antioxidant defense: Cu/Zn-SOD, GPx, CAT, GCL, Nrf2, Keap1, TOR, S6K1 and CK2 mRNA levels

**Figure 3:**
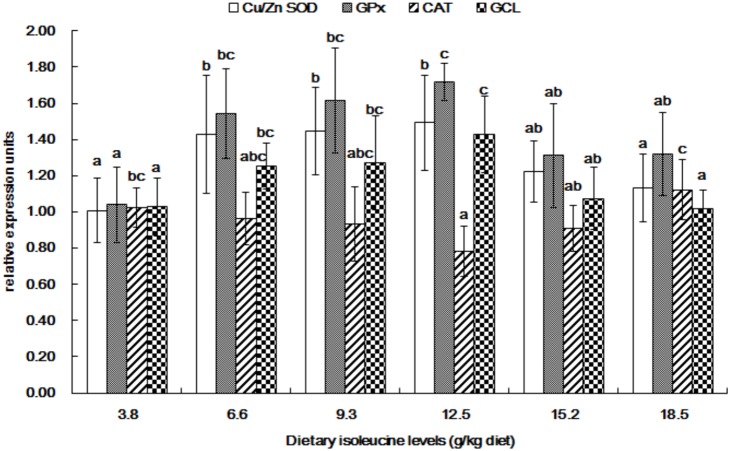
Effects of dietary Ile on Cu/Zn-SOD, GPx, CAT and GCL expression in grass carp muscle. Values are means with standard deviations represented by vertical bars (n = 6). Different letter above bars indicated significant difference among treatments (*P*<0.05). Cu/Zn-SOD, copper/zinc superoxide dismutase; GPx, glutathione peroxidase; CAT, catalase; GCL, glutamate-cysteine ligase.

**Figure 4:**
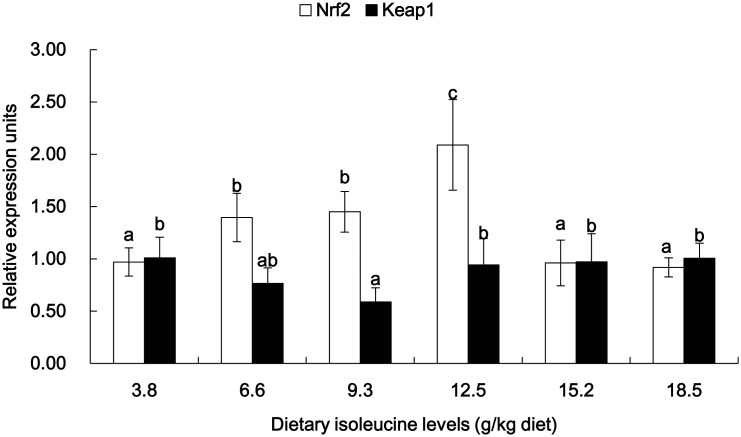
Effects of dietary Ile on Nrf2 and Keap1 expression in grass carp muscle. Values are means with standard deviations represented by vertical bars (n = 6). Different letter above bars indicated significant difference among treatments (*P*<0.05). Nrf2, NF-E2-related factor 2; Keap1, Kelch-like- ECH-associated protein 1.

**Figure 5:**
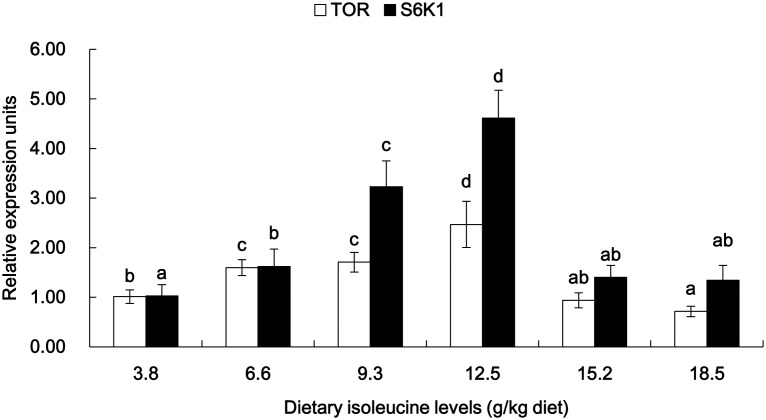
Effects of dietary Ile on TOR and S6K1 expression in grass carp muscle. Values are means with standard deviations represented by vertical bars (n = 6). Different letter above bars indicated significant difference among treatments (*P*<0.05). TOR, target of rapamycin; S6K1, ribosomal S6 protein kinase 1.

**Figure 6:**
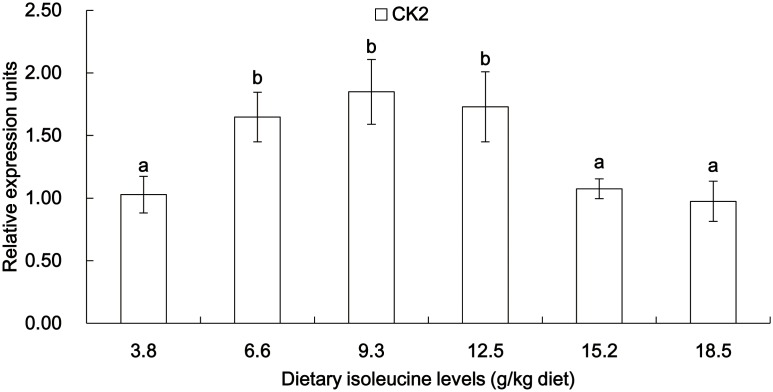
Effects of dietary Ile on CK2 expression in grass carp muscle. Values are means with standard deviations represented by vertical bars (n = 6). Different letter above bars indicated significant difference among treatments (*P*<0.05). CK2, casein kinase 2.

The gene expressions of Cu/Zn-SOD, GPx, CAT, GCL, Nrf2, Keap1, TOR, S6K1 and CK2 in the muscle of young grass carp fed diets containing graded levels of Ile are shown in Figs. 3–6. The mRNA levels of Cu/Zn-SOD, GPx and GCL gradually improved with the increase of dietary Ile levels up to 12.5 g/kg diet and then significantly depressed (*P*<0.05) (Fig. 3). The CAT mRNA levels decreased as dietary Ile levels up to 12.5 g/kg diet and subsequently increased (*P*<0.05) (Fig. 3). The Nrf2 mRNA levels were the highest for fish that were fed 12.5 g Ile/kg diet, followed by 6.6 and 9.3 g Ile/kg diet (*P*<0.05) (Fig. 4), and no significant differences were found among the other groups (*P*>0.05) (Fig. 4). Keap1 mRNA levels were depressed with higher dietary Ile levels up to 9.3 g Ile/kg diet and subsequently increased (*P*<0.05) (Fig. 4), whereas higher Ile levels resulted in a plateau-related response (*P*>0.05) (Fig. 4). The TOR and S6K1 mRNA levels gradually increased with increasing Ile levels up to 12.5 g Ile/kg diet and after that decreased (*P*<0.05) (Fig. 5). The fish that were fed diets with 6.6 g Ile/kg, 9.3 g Ile/kg and 12.5 g Ile/kg diet showed higher CK2 mRNA levels than other groups (*P*<0.05) (Fig. 6).

## Discussion

### Ile improved growth performance and muscle nutrient deposition of fish

The acclimatization of laboratory animals aims to minimize stress associated with transportation and to restore their physiological homeostasis, thus eliminating the effect of physiological changes on the research results [39]. In terrestrial animal, the majority of physiological changes in the immune and endocrine systems take 1 to 7 days to normalize [39]. According to the reports, fish have been found to restore physiological homeostasis in two weeks after being transported [40]. In studies investigating the effect of dietary nutrients on fish flesh quality, fish were typically acclimated to the experimental condition for two weeks [41]–[44]. Therefore, in the present study, we chose two weeks as the acclimatization period. Young grass carp that were fed the Ile-deficient diet (3.8 g Ile/kg diet) obtained a poor PWG, which was consistent with the reports for juvenile Indian major carp (*Cirrhinus mrigala*) [30]. The weight gain of fish, in large part, depends on the protein and fat deposition in muscle [2], [45]. The data have demonstrated that the muscle protein and fat contents were lower in the Ile-deficient group and gradually increased with increasing dietary Ile concentrations up to 12.5 g/kg diet, and subsequently decreased, showing that optimal Ile can enhance protein and fat deposition in fish muscle. Furthermore, the protein deposition is associated with amino acid metabolism in fish [30]. Ammonia is the major product of fish amino acid catabolism [28]. In this study, Ile deficiency led to the highest plasma ammonia content in young grass carp; with optimal dietary Ile supplementation, the plasma ammonia content declined, suggesting that appropriate Ile could attenuate amino acid catabolism, resulting in higher amino acid utilization and protein deposition in fish muscle. Moreover, research has indicated that the level of certain EAAs in fish muscle was used as an index of dietary amino acid status [44]. Our results showed that Ile deficiency caused a drop in the total EAAs level in young grass carp muscle and that 6.6–15.2 g Ile/kg diet supplementation could inhibit the decrease, indicating that optimal Ile supplementation simulated a balanced amino acid condition in diet and enhanced protein deposition in fish muscle. Additionally, this study showed that young grass carp that were fed excess Ile (18.5 g Ile/kg diet) had lower leucine concentration in the muscle than fish that were fed 6.6–15.2 g Ile/kg diet, which highlighted an antagonist effect of excess Ile on the leucine level in fish muscle. Similarly, antagonism effects between Ile and leucine were observed in lake trout (*Salvelinus namaycush*) and rainbow trout (*Oncorhynchus mykiss*) [6]. The data showed that optimum Ile supplementation could promote deposition of nutrients (fat, protein and EAAs) in young grass carp muscle. Apart from the muscle nutrient composition, the physical quality indicators, such as pH value, firmness and WHC play a key role in consumer acceptance [46].

### Ile improved flesh quality of fish

Muscle pH value is considered to be an important flesh quality parameter and low initial pH post-mortem could be responsible for the deterioration of flesh quality in fish [34]. In the present study, the muscle pH was lower in the Ile-deficient group and significantly increased with higher dietary Ile levels up to 6.6–12.5 g/kg diet, which noted that appropriate Ile improved the muscle pH to prevent flesh quality deterioration in fish. The suppression of lactate accumulation in fish muscle by Ile may contribute to the improvement of muscle pH. Studies on Atlantic salmon implied that the decrease of muscle pH value was ascribed to the lactate accumulation [10]. Our study showed that Ile deficiency induced an elevation of lactate concentration in young grass carp muscle and that when supplement Ile was administered at an adequate level, the elevation was reversed. Additionally, a negative correlation was observed between the pH value and lactate concentration (r = −0.810, *P* = 0.051), which suggested that Ile may have partially improved the muscle pH value by reducing lactate concentration in fish. The inhibition effect of Ile on lactic acid accumulation may be related to the reduced lactate dehydrogenase release in fish muscle. Lactate dehydrogenase is a key enzyme in lactic acid biosynthesis during anaerobic respiration [47]. The first step in Ile catabolism is transamination, which leads to the production of KMV [48]. KMV has been reported to attenuate lactate dehydrogenase release in rat neuron cells [16]. However, whether Ile suppressed the lactic acid accumulation by decreasing the lactate dehydrogenase release in fish muscle warrants additional investigation. The muscle pH value decline was typically accompanied by muscle softening and water loss in fish [49].

Muscle firmness and WHC are two important flesh quality parameters and the decline of these parameters is usually regarded as an indicator of quality loss in fish fillet [34]. In this study, Ile deficiency (3.8 g Ile/kg diet) and excess (18.5 g Ile/kg diet) led to a drop in young grass carp muscle shear force by approximately 20% and 30% compared with 9.3 g Ile/kg diet group, respectively. Meanwhile, Ile deficiency and excess caused an increase in muscle cooking loss to nearly 24% and 26% above 12.5 g Ile/kg diet group, respectively. The relationships between shear force, cooking loss and dietary Ile levels were described by the following quadratic equations, respectively: Y_shear force_ = −0.006x^2^+0.13x+1.440, R^2^ = 0.830, *P* = 0.070; Y_cooking loss_ = 0.115x^2^−3.108x+33.402, R^2^ = 0.854, *P* = 0.056, indicating that appropriate Ile supplementation could enhance muscle firmness and WHC, thereby improving fish flesh quality. Ile-enhanced firmness and WHC in fish muscle may be attributed to the decreased cathepsin activities. Cathepsin B and L played a key role in fish muscle degradation [35]. The muscle degradation could lead to the fillet softening in Atlantic salmon [34] and fillet water loss in sardine (*Sardine pilchardus*) [13]. In our study, Ile deficiency and excess induced the enhancement of cathepsin B and L activities in young grass carp muscle, whereas 9.3–12.5 g Ile/kg supplementation could prevent this enhancement, suggesting that optimal Ile could reduce activities of cathepsin B and L in fish muscle. As shown in Table 7, the correlation analysis indicated that muscle shear force was negatively and cooking loss was positively related to the cathepsin B and L activities, demonstrating that Ile enhanced fish muscle firmness and WHC in part by decreasing cathepsin B and L activities. Additionally, the enhanced firmness and WHC in this study may be observed because Ile elevated collagen content in fish muscle. It was implied that the high collagen content contributed to muscle firmness in Atlantic salmon [50]. Ofstad et al. [51] demonstrated that collagen glued together the muscle fibers and fiber bundles, which contributed to water holding of fish muscle. In the present study, compared with Ile deficiency and excess, optimal dietary Ile supplementation increased collagen content in young grass carp muscle. The correlation analysis showed that the muscle shear force was positively and cooking loss was negatively correlated to the collagen content (Table 7), suggesting that Ile-enhanced firmness and WHC may be in part attributed to the increased collagen content in fish muscle. Moreover, the positive effect of Ile on firmness may be correlated with the increased glutamic acid content in fish muscle. Larsson et al. [8] indicated that glutamate supplementation resulted in firmer fillets in Atlantic salmon. In this study, young grass carp that were fed Ile deficient and excessive diets had lower glutamic acid contents in the muscle than fish that were fed 6.6–15.2 g Ile/kg diet, demonstrating that appropriate Ile elevated glutamic acid content in fish muscle. As shown in Table 7, a positive correlation was observed between muscle shear force and glutamic acid content, which suggested that Ile improved fish muscle firmness in part by increasing the glutamic acid content in fish muscle. All together, the data showed that appropriate dietary Ile could improve grass carp flesh quality. Several studies have demonstrated that the ROS-caused oxidative damage of lipids and proteins was suggested to be a major cause of flesh quality loss in fish [31].

**Table 7:** Correlation coefficients between shear force, cooking loss and cathepsin B and L activities, hydroxyproline, Glu contents in grass carp muscle.

	shear force	*P* value	cooking loss	*P* value
Cathepsin B	−0.876	<0.05	+0.854	<0.05
Cathepsin L	−0.704	0.118	+0.828	<0.05
Hydroxyproline	+0.920	<0.01	0.655	0.158
Glu	+0.685	0.134	nd	nd

nd = correlation not-determined.

### Ile enhanced antioxidant defense and regulated antioxidant enzyme gene expression in fish muscle

O_2_^•−^ and •OH are two oxygen free radicals strongly involved in oxidative damage, which primarily shows as lipid peroxidation and protein oxidation [12], [14]. In the present study, relative to Ile deficiency and excess, appropriate dietary Ile supplementation enhanced O_2_^•−^-scavenging ability (indicated by ASA capacity) and •OH-scavenging ability (indicated by a-HR capacity) and decreased MDA and PC contents in young grass carp muscle, supporting that optimal Ile promoted the removal of free radicals to alleviate oxidative damage in fish muscle. Oxygen free radical removal is dependent on non-enzymatic compounds, such as GSH, and antioxidant enzymes, such as Cu/Zn-SOD, GPx and CAT in fish [12]. GCL is the rate-limiting enzyme in GSH synthesis [52]. In our study, Ile deficiency and excess induced a decline of GSH content in the muscle of young grass carp, whereas optimal Ile supplementation could reverse this decline; likewise, the mRNA level of GCL followed a similar pattern with GSH content. The correlation analysis showed a significant positive correlation between GSH content and GCL mRNA level (r = +0.864, *P*<0.05) in the muscle of young grass carp, indicating that Ile improved muscle GSH content in fish muscle in part by promoting the mRNA expression of GCL. This study showed that Ile deficiency and excess caused decreased Cu/Zn-SOD and GPx activities in young grass carp muscle, and appropriate dietary Ile could inhibit the decrease. Positive correlations were observed between ASA and Cu/Zn-SOD activity (r = +0.888, *P*<0.05), as well as a-HR and GPx activity (r = +0.650, *P* = 0.162), demonstrating that Ile-enhanced radical-scavenging ability may be ascribed to the improvement of Cu/Zn-SOD and GPx activities in fish muscle. As shown in Table 8, the correlation analysis indicated that muscle shear force was positively correlated with GSH content, the activities of Cu/Zn-SOD and GPx and negatively correlated with MDA content, PC content, whereas the muscle cooking loss was positively related to MDA content, PC content and negatively related to GSH content, GPx activity. All of these data provided a likely explanation for Ile to improving fish flesh quality by enhancing antioxidant defense in young grass carp muscle.

**Table 8:** Correlation coefficients between shear force, cooking loss and malondialdehyde (MDA), protein carbonyl (PC), anti-superoxide anion (ASA), anti-hydroxy radical (a-HR) activities, glutathione (GSH) content, copper/zinc superoxide dismutase (Cu/Zn-SOD), glutathione peroxidase (GPx) activities in grass carp muscle.

	shear force	*P* value	cooking loss	*P* value
MDA	−0.769	0.074	+0.915	<0.05
PC	−0.703	0.119	+0.672	0.143
GSH	+0.749	0.086	−0.932	<0.01
Cu/Zn-SOD	+0.718	0.108	−0.389	0.446
GPx	+0.923	<0.01	−0.773	0.071

The antioxidant defense in fish, in large part, relies on the activities of antioxidant enzymes [14]. Previous studies have demonstrated that antioxidant enzyme activities were closely associated with their gene expressions [18]. Our data demonstrated that the Cu/Zn-SOD and GPx mRNA levels in muscle were lower in young grass carp that were fed Ile deficiency and excess diets compared with the adequate dietary Ile-supplemented group, which was positively correlated to their respective enzyme activities (r = +0.591, *P* = 0.217; r = +0.790, *P* = 0.062). The results suggest that Ile-improved Cu/Zn-SOD and GPx activities may be attributed to the up-regulation of their mRNA expressions in fish muscle. Interestingly, in the present study, Ile deficiency and excess enhanced the activity and mRNA level of CAT in young grass carp muscle. The explanation may be that Ile deficiency and excess induced the increase of H_2_O_2_ in fish muscle. Ile is the precursor of KMV and Ile deficiency could cause the depletion of KMV in fish muscle [6]. It was implied that the lack of KMV resulted in an elevated level of H_2_O_2_ in human skin fibroblasts [16]. Bridi et al. [53] suggested that excessive Ile accumulation increased the production of H_2_O_2_ in rat cerebral cortex. Additionally, studies in rat neonatal ventricular myocytes have shown that high concentrations of H_2_O_2_ can induce the transcriptional activation of CAT, which in turn can led to the improvement of its activity [54]. Hence, the elevated CAT activity and mRNA expression by dietary Ile deficiency and excess may be related to the increase of H_2_O_2_, which warrants additional investigation in fish muscle.

### Ile regulated antioxidant-related signaling molecule expressions in fish muscle

Nrf2 is a major transcription factor that regulates gene expression of antioxidant enzymes [19]. Several studies have indicated that the up-regulation of Nrf2 gene expression could increase the Cu/Zn-SOD mRNA level in human mesenchymal stem cells [55] and the GPx mRNA level in mice lung [56]. In the current study, Ile deficiency and excess caused a down-regulation of the Nrf2 mRNA expression level in young grass carp muscle, and optimal dietary Ile could reverse this down-regulation. The correlation analysis indicated that the mRNA levels of Cu/Zn-SOD (r = +0.836, *P*<0.05) and GPx (r = +0.849, *P*<0.05) were positively related to the Nrf2 mRNA level, suggesting that the enhanced Cu/Zn-SOD and GPx mRNA expressions by Ile may be in part ascribed to the up-regulation of Nrf2 mRNA expression in fish muscle. To the best of our knowledge, the nuclear translocation of Nrf2 was essential for transcriptional activation of antioxidant enzyme genes in mice [57]. Keap1, a cytosolic Nrf2-binding protein, bridges Nrf2 to Cul3-based E3 ubiquitin ligase for proteasome degradation [58]. It was shown that the down-regulation of Keap1 gene expression in murine lung promoted Nrf2 to move from cytoplasm to the nucleus, resulting in the enhancement of Cu/Zn-SOD and GPx mRNA expressions [59]. In the present study, compared with 9.3 g Ile/kg diet, deficiency and excess of Ile resulted in an up-regulation of Keap1 mRNA expression in young grass carp muscle, suggesting that optimal Ile may induce the nuclear translocation of Nrf2 to enhance Cu/Zn-SOD and GPx gene expressions by down-regulating the expression of Keap1 mRNA in fish muscle.

Additionally, Nrf2 expression is regulated by its upstream signaling molecules, such as TOR. It was reported that TOR together with its target, ribosomal S6 protein kinase1 (S6K1), could stimulate Nrf2 expression in human brain endothelial cells [60]. Furthermore, CK2 emerged as a key regulator in the activation of TOR expression in human lung carcinoma cells [21]. In our study, we showed that Ile deficiency and excess led to a down-regulation of TOR, S6K1 and CK2 mRNA levels in young grass carp muscle and that with appropriate dietary Ile supplementation, the down-regulation was restored. The correlation analysis showed that the Nrf2 mRNA level was positively correlated to the mRNA levels of TOR (r = +0.990, *P*<0.001) and S6K1 (r = +0.936, *P*<0.05), whereas a positive correlation was observed between the TOR mRNA level and the CK2 mRNA level (r = +0.870, *P*<0.05), implying that Ile-induced up-regulation in Nrf2 mRNA expression was due in part to the enhancement of TOR, S6K1 and CK2 mRNA expressions in fish muscle. However, additional studies are warranted to explore the detailed manner in which Ile regulates the expression of these signaling molecules of the Nrf2 pathway in fish.

### Ile requirements of young grass carp

The dietary Ile requirements of young grass carp (253.4–660.8 g) estimated from muscle cooking loss and PWG using quadratic regression analysis were 11.1 g/kg and 12.2 g/kg diet, corresponding to 36.0 g/kg and 39.3 g/kg protein, respectively. Similar requirements may result from the optimal dietary Ile enhanced protein synthesis in fish muscle. Periago et al. [61] implied that muscle protein, such as sarcoplasmic protein, played an important role in fish muscle water holding. Several studies have confirmed that protein deposition in muscle tissue was the foundation of fish growth [34]. In our study, optimal Ile supplementation could promote protein deposition in the muscle of young grass carp, which may interpret the similar requirements estimated from muscle cooking loss and PWG. The effect of dietary Ile on muscle protein properties correlated with flesh quality should be evaluated in the future.

### Conclusions

The present study demonstrated that both dietary Ile deficiency and excess led to flesh quality deterioration (softening, water loss and pH decline) of fish and that when supplement dietary Ile was administered at an appropriate level, the flesh quality deterioration was restored. Appropriate dietary Ile supplementation improved flesh quality in part by suppressing lipid peroxidation and protein oxidation, which may be attributed to the enhancement of GSH content and Cu/Zn-SOD and GPx activities. The results of mRNA expressions of Cu/Zn-SOD and GPx might go further to support Ile-increased Cu/Zn-SOD and GPx activities. Moreover, the results of mRNA expressions of signaling molecules (Nrf2, Keap1, TOR, S6K1 and CK2) involved in the Nrf2 pathway revealed the potential beneficial effect of dietary Ile on Cu/Zn-SOD and GPx gene expressions. All of the data provided a portion of theoretical basis for developing Ile as an effective natural antioxidant to improve flesh quality in fish. Nevertheless, additional investigation must be conducted to study the detailed mechanisms by which dietary Ile improves flesh quality of fish. Meanwhile, the dietary Ile requirement based on muscle cooking loss of young grass carp (253.4–660.8 g) was 11.1 g/kg diet, corresponding to 36.0 g/kg protein, which was approximate to the Ile requirement for PWG (12.2 g/kg diet or 39.3 g/kg protein).

## Supporting Information

S1 Table **Real-time primer sequences, thermocycling conditions and accession numbers for 18S rRNA, elongation factor 1 alpha (EF1-α), beta-actin (β-actin) and glycer-aldehyde-3-phosphate dehydrogenase (GAPDH) genes.**
(DOCX)

S2 Table **The cycle threshold (Ct) values of each housekeeping gene in the muscle of young grass carp fed diets with graded levels of Ile (g/kg diet).**
(DOCX)

S3 Table **Ranking of four selected internal control gene stability values in the muscle of young grass carp fed diets with graded levels of Ile (g/kg diet).**
(DOCX)
