# Supplementary material for: Flesh Quality Loss in Response to Dietary Isoleucine Deficiency and Excess in Fish: A Link to Impaired Nrf2-Dependent Antioxidant Defense in Muscle
Source: PLoS One. 2014 Dec 16;9(12):e115129. doi: 10.1371/journal.pone.0115129 (PMC4267783; doi:10.1371/journal.pone.0115129)
Supplement: S1 Table — Real-time primer sequences, thermocycling conditions and accession numbers for 18S rRNA, elongation factor 1 alpha (EF1-α), beta-actin (β-actin) and glycer-aldehyde-3-phosphate dehydrogenase (GAPDH) genes. (DOCX) [file pone.0115129.s001.docx]

**Table S1**

Real-time primer sequences, thermocycling conditions and accession numbers for 18S rRNA, elongation factor 1 alpha (EF1-α), beta-actin (β-actin) and glycer-aldehyde-3-phosphate dehydrogenase (GAPDH) genes.

| Gene | Sequences of primers | Thermocycling conditions | Accession number |
| --- | --- | --- | --- |
| 18S rRNA |  |  | EU047719 |
| Forward | 5^/^- ATTTCCGACACGGAGAGG -3^/^ | 95 °C 30 s, 40 cycles of 95 °C 5 s, 59.0 °C 30 s and 72 °C 30 s |  |
| Reverse | 5^/^- CATGGGTTTAGGATACGCTC -3^/^ |  |  |
| EF1-α |  |  | GQ266394 |
| Forward | 5^/^- CGCCAGTGTTGCCTTCGT -3^/^ | 95 °C 30 s, 40 cycles of 95 °C 5 s, 59.0 °C 30 s and 72 °C 30 s |  |
| Reverse | 5^/^- CGCTCAATCTTCCATCCCTT -3^/^ |  |  |
| GAPDH |  |  | GQ266395 |
| Forward | 5^/^- GTTACAAGGGAGAAGTTCACCAT -3^/^ | 95 °C 30 s, 40 cycles of 95 °C 5 s, 61.4 °C 30 s and 72 °C 30 s |  |
| Reverse | 5^/^- CCGGTAGACTCGACTACATACAG -3^/^ |  |  |
| β-actin |  |  | M25013 |
| Forward | 5^/^- GGCTGTGCTGTCCCTGTA-3^/^ | 95 °C 30 s, 40 cycles of 95 °C 5 s, 61.4 °C 30 s and 72 °C 30 s |  |
| Reverse | 5^/^- GGGCATAACCCTCGTAGAT-3^/^ |  |  |
