# Supplementary material for: Flesh Quality Loss in Response to Dietary Isoleucine Deficiency and Excess in Fish: A Link to Impaired Nrf2-Dependent Antioxidant Defense in Muscle
Source: PLoS One. 2014 Dec 16;9(12):e115129. doi: 10.1371/journal.pone.0115129 (PMC4267783; doi:10.1371/journal.pone.0115129)
Supplement: S2 Table — The cycle threshold (Ct) values of each housekeeping gene in the muscle of young grass carp fed diets with graded levels of Ile (g/kg diet). (DOCX) [file pone.0115129.s002.docx]

**Table S2**

The cycle threshold (Ct) values of each housekeeping gene in the muscle of young grass carp fed diets with graded levels of Ile (g/kg diet).

| Ile | 0.7 | 1.7 | 3.1 | 4.0 | 5.2 | 6.1 |
| --- | --- | --- | --- | --- | --- | --- |
| 18S rRNA | 12.39 ±0.31 | 12.77 ±0.36 | 12.84 ±0.92 | 12.27 ±0.44 | 12.02±1.21 | 12.19 ±1.26 |
| EF1-α | 26.25 ±0.58 | 25.70 ±1.46 | 25.99 ±0.28 | 25.61 ±0.39 | 25.80 ±1.01 | 26.29 ±0.83 |
| β-actin | 20.96 ±0.65 | 21.35 ±0.85 | 21.34 ±1.22 | 21.16 ±0.69 | 21.27 ±0.42 | 21.20 ±0.52 |
| GAPDH | 28.29 ±1.17 | 28.88 ±2.03 | 28.85 ±0.94 | 28.35 ±0.61 | 28.46±0.97 | 28.08±1.47 |

Values are means ± SD (n=6).
