# Supplementary material for: Flesh Quality Loss in Response to Dietary Isoleucine Deficiency and Excess in Fish: A Link to Impaired Nrf2-Dependent Antioxidant Defense in Muscle
Source: PLoS One. 2014 Dec 16;9(12):e115129. doi: 10.1371/journal.pone.0115129 (PMC4267783; doi:10.1371/journal.pone.0115129)
Supplement: S3 Table — Ranking of four selected internal control gene stability values in the muscle of young grass carp fed diets with graded levels of Ile (g/kg diet). (DOCX) [file pone.0115129.s003.docx]

**Table S3**

Ranking of four selected internal control gene stability values in the muscle of young grass carp fed diets with graded levels of Ile (g/kg diet).

| Gene name | geNorm M value  (order) | NormFinder stability  value (order) | Ranking  order |
| --- | --- | --- | --- |
| 18S rRNA | 0.320 (2) | 0.127 (2) | 2 |
| EF1-α | 0.453 (4) | 0.292 (4) | 4 |
| β-actin | 0.308 (1) | 0.071 (1) | 1 |
| GAPDH | 0.324 (3) | 0.158 (3) | 3 |

The input data for geNorm and NormFinder were 2^-ΔCT^ values.
